# Treatment of Rosacea using acupuncture for improving the local skin microcirculation: A case report

**DOI:** 10.1097/MD.0000000000011931

**Published:** 2018-08-24

**Authors:** Yacen Gao, Weipeng Lin, Sisi Zhou, Guoqi Shi, Jun He, Yongjun Chen

**Affiliations:** a South China Research Center for Acupuncture and Moxibustion, Medical College of Acu-Moxi and Rehabilitation, Guangzhou University of Chinese Medicine; b Rehabilitation Center, the First Affiliated Hospital of Guangzhou University of Chinese Medicine, Guangzhou, China.

**Keywords:** acupuncture, microcirculation, Rosacea

## Abstract

**Rationale:**

Rosacea is an irritating disease that affects patients’ health and life quality. The current treatments for rosacea have limited efficacy and are generally not satisfying most patients. This report presents a patient diagnosed with rosacea who was treated with acupuncture to a satisfactory effect. Laser Doppler was used to measure the local blood perfusion of the nose before, during, and after acupuncture treatment. The Dermatology Life Quality Index (DLQI) was used to measure the impact of rosacea on the quality of the patient's life.

**Patient concerns:**

A 52-year-old woman had been diagnosed with rosacea 18 months before this study. She had tried medical treatments in other hospitals with metronidazole cream, antifungal drugs, and steroidal ointments, but the effect was poor and limited.

**Diagnoses:**

In this study, the diagnosis of rosacea (stage I, subtype Erythematotelangiectatic) was made by a dermatologist according to physical examination).

**Interventions:**

The patient's treatment included a half-hour of acupuncture 3 times per week.

**Outcomes:**

The patient experienced significant improvements in the region around the nose after 3 sessions of acupuncture treatment within the first week and reported that there was no relapse for 6 months after acupuncture treatment. The perfusion of blood flow was redistributed during and after acupuncture treatment according to laser Doppler measurements. The patient's DLQI score substantially improved. The patient was generally satisfied with the acupuncture treatment.

**Lessons:**

The results suggested that acupuncture might be an alternative therapy for facial localized rosacea. As well, acupuncture may be effective in treating rosacea through redistributing micro-circulation of blood at the localized area of effect. The overall costs of the rosacea treatment may be reduced, provided that this therapy is demonstrated to be effective in future controlled studies.

## 1 Introduction

Rosacea is a skin disease that typically result in facial redness, swelling, itching, pimples, pustules, increased sebum excretion, and superficial dilated blood vessels.^[1]^ The appearance of symptoms of rosacea can cause negative emotions such as embarrassment, anxiety, and low self-esteem in patients, which severely affects their quality of life.^[2]^

The current therapies for rosacea include medication and surgery; however, most patients remain dissatisfied with the outcomes of such treatments. The therapeutic effectiveness of medication for rosacea is limited and patients are frequently impacted by the side effect of medication, such as epidermal desquamation scrape, erythema, xerosis cutis, and burning sensation, along with the high risk of relapse after medication treatment.^[3–5]^ Surgical treatments are invasive and have potential risks of wound forming and airway obstruction during the surgery, which makes this an unpreferred course of treatment in mild to moderate rosacea. Also, the recovery time from surgery may last several months to even years, and the potential complications such as scar formation, alar notching, and alar fistula may occur after surgery. The success of the surgery largely depends on doctors’ skills,^[6–8]^ and despite the variety of therapeutic options, none are completely curative. Therefore, further research on the pathophysiology of rosacea is required in order to find more targeted and efficacious treatment options.^[9]^

Acupuncture is a method of treatment originating from traditional Chinese medicine, which has been used to treat facial and skin diseases since ancient times. Modern studies suggested that acupuncture is a potentially effective alternative therapy for the treatment of skin disease such as atopic eczema^[10]^ and psoriasis.^[11]^ In this case, a female patient diagnosed with rosacea and dissatisfied with conventional medical treatment was treated successfully with acupuncture. Furthermore, we used the laser Doppler to measure the local blood perfusion of the nose before, during, and after acupuncture treatment. To the best of our knowledge, this is the first report that addresses treatment of rosacea with acupuncture and makes use of an analysis of blood perfusion of the nose before, during, and after acupuncture treatment.

## 2 Case description

The patient is a 52-year-old woman, who first presented to our clinic on November 30, 2016, with complaints of redness, red rash, and increased sebum excretion of the nose. These symptoms were sometimes accompanied by repeated occurrences of itching, papules, and pustules, and had been present for 1 year. Her symptoms were triggered by unknown causes and worsened after eating any greasy food. She was diagnosed with rosacea and tried medical treatments in other hospitals with metronidazole cream, antifungal drug, and steroidal ointments, but the therapeutic effect was poor. She made the decision to try acupuncture; she ceased receiving any medical treatment 1 month before she received the acupuncture treatment on March 13, 2017.

## 3 Examination

**Figure 1:**
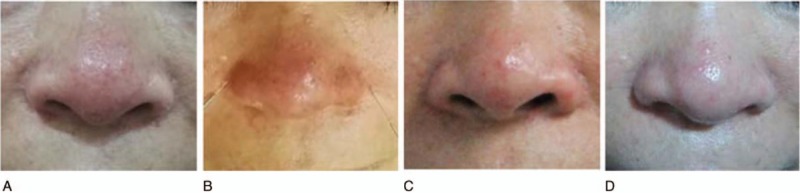
The patient's appearance before, during, and after acupuncture treatment. (A) Before the acupuncture treatment. (B) During the acupuncture treatment. (C) One week after acupuncture treatment started. (D) Six months after acupuncture treatment.

Her medical history indicated a total hysterectomy for uterine fibroids in 2009, with good recovery. She stated no any history of drug or food allergy and did not have hypertension, diabetes, hepatitis, tuberculosis, or any other conditions or medical history. The physical examination revealed diffused erythema, papules, and telangiectasia of the nose (Fig. 1A, B). The life quality of the patient was assessed by Dermatology Life Quality Index (DLQI) before acupuncture treatment and the score was 15 on a scale of 0 to 30, which indicated her facial rosacea had decreased her quality of life.

## 4 Diagnosis

The diagnosis of rosacea (stage I, subtype Erythematotelangiectatic) was made by a dermatologist according to a physical examination.

## 5 Intervention

The patient was treated with acupuncture in 3 sessions for 1 week. The study procedures were explained to the patient and informed consent was obtained.

**Figure 2:**
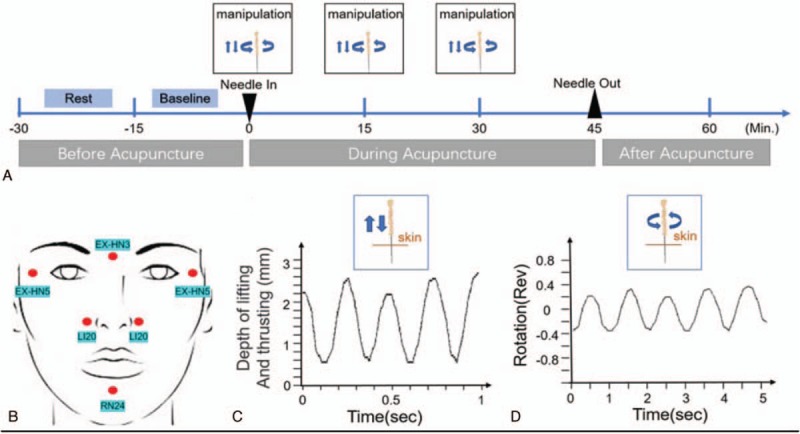
Procedure of acupuncture and operation parameters. (A) Schematic protocol of acupuncture manipulation (3 times 1 week). (B) Locations of acupuncture points. (C) Operation parameters for lifting and thrusting. (D) Operation frequency of twirling and rotation.

The acupoints selected in this case are generally used for facial skin diseases and are based on acupuncture local acupoint-selection principles. The patient was in a supine position during acupuncture treatment. After the facial skin was sterilized, the acupuncture needles (diameter 0.3 mm; length 25 mm) were inserted into the acupoints of Yintang (EX-HN3), bilateral Taiyang (EX-HN5), bilateral Yingxiang (LI20), and Chengjiang (CV 24) (Figs. 1B and 2B Fig. 2). The inserted depth was 1 to 3 mm. As shown in Fig. 2, the uniform acupuncture manipulations included lifting and thrusting, twirling and rotating the needles, which were repeated 3 times every 15 minutes (Fig. 2A). As shown in Fig. 2C and D, the depth and frequency of operation parameters were analyzed by acupuncture manipulation parameter tester (ATP-II; Shanghai Shangyou Medical Equipment Co. Ltd, Shanghai, China). The “deqi” (the patient's feeling of fullness, heaviness, dull aching due to the needles) was obtained. The needles were retained in acupoints for another 15 minutes without any operation. The patient was instructed not to use any medication during and after the treatment of acupuncture.

## 6 Follow-up and outcome

The patient experienced significant improvement in erythema and papules of the nose, and a complete relief of symptoms relating to telangiectasia and itching after 3 sessions of acupuncture treatment within 1 week (Fig. 1C).

The follow-up time period was 6 months after the initial treatment, from May 17, 2017, to November 17, 2017. The patient reported that there was no relapse of the rosacea for 6 months after acupuncture treatment (Fig. 1D) and was generally satisfied with the acupuncture treatment. The quality of life for the patient was assessed by DLQI at periods of 1 week and 6 months after acupuncture treatment. Both DLQI score was 0, which showed that the patient was experiencing a great improvement in her quality of life. There were no adverse or unanticipated events during the treatment or outcome periods.

## 7 Blood perfusion imaging analysis

One of the most common conditions associated with rosacea is dilation of blood vessels under the skin, which causes persistent flushing and redness.^[1]^ To evaluate the characteristics of skin blood flow around the patient's nose, we used laser Doppler perfusion imaging before, during, and after the acupuncture treatment.

**Figure 3:**
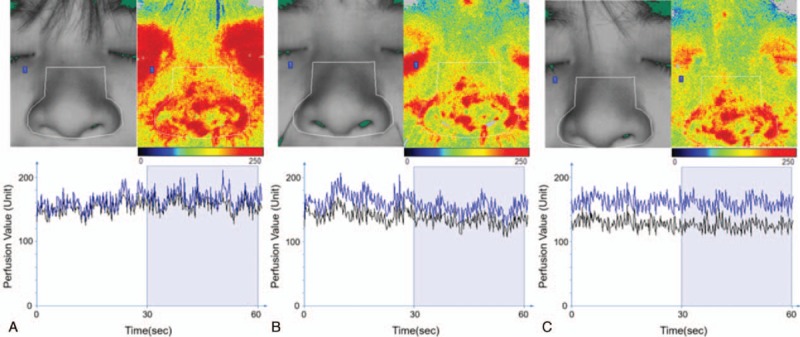
The perfusion of the facial and nasal area before (A), during (B), and after (C) the acupuncture treatment. The color of the upper right pictures represents the strength of perfusion under the skin, and the red area represents the richness of perfusion. The black line of the lower panel represents the mean perfusion value of the facial area, and the blue line represents the mean perfusion value of the selected nasal area (indicated as white line frames in the facial photos of the upper panel). The mean perfusion value in 30 s was chosen after the value became stable.

Laser-Doppler (PERIMED PSI-ZR; Perimed AB, Jarfalla, Sweden) was used to evaluate the skin perfusion changes before, during, and after acupuncture stimulus. As shown in Fig. 3, a 1229 mm^2^ region of nasal area and a 4159 mm^2^ region of facial area covering the nose were measured. The average data acquisition measuring time was 2 minutes.

As laser Doppler perfusion imaging shows, the mean perfusion value of the facial area was 151 units, and the mean perfusion value of the nasal area was 167 units before the acupuncture treatment (Fig. 3A). During the treatment, the perfusion values decreased, and the mean perfusion of the facial area was 129 units, while the mean perfusion of the nasal area was 141 units (Fig. 3B). We found that the perfusion of blood flow was redistributed 30 minutes after withdrawal of the needle, and the mean perfusion of the facial area was 129 units, and the nasal area was 158 units (Fig. 3C). This result suggested that the acupuncture treatment might be effective in treating rosacea through blood redistribution and micro-circulation of local skin area.

## 8 Discussion

The current case report shows that patients with erythematotelangiectatic rosacea might benefit from treatment with acupuncture, based on the results of the subject patient's relief from symptoms of erythema, papules, itching, and telangiectasia after acupuncture treatment, and without relapse in the following 6 months. The patient was treated with acupuncture for 1 week in three total sessions without any side effects or relapse for 6 months. Conventional medical treatments such as topical and oral antibiotics or retinoids may have severe side effects such as epidermal desquamation scrape, erythema, xerosis cutis, and burning sensation^[3–5]^ and surgical treatments are invasive and have potential risks of wound forming and airway obstruction during the surgery and potential complications such as scar formation, alar notching, alar fistula, and the recovery time of surgery can last several months or even years. The outcome of surgery largely depends on the skills of individual surgeons.^[6–8]^ In comparison to these conventional treatments, administration of acupuncture relieves symptoms for an extended duration and presents with no side effects.

The pathophysiology of rosacea remains largely unclear; however, vascular abnormalities might contribute significantly to the symptoms of rosacea.^[1]^ For example, facial blood flow reversal in response to overheating is diminished in patients with rosacea.^[12]^ In recent years, laser Doppler perfusion imaging has been used to evaluate the characteristics of vascular activity under the skin and the degree of severity of skin diseases.^[13–15]^ Laser Doppler perfusion imaging is also a tool to study the mechanism of acupuncture's effectiveness in treating disease.^[16,17]^ Acupuncture stimulation increases the local blood flow around the site of stimulation,^[16]^ and repeated acupuncture manipulations enhance microcirculatory perfusion of local Hegu (LI4) acupoint area.^[17]^ A recent study used laser Doppler to evaluate the skin perfusion after acupuncture in atopic eczema patients, and the result showed that the mean perfusion value was significantly smaller in acupuncture group, than nonacupuncture group.^[10]^

Acupuncture on alternating days or 3 times per week is common and effective in treating skin diseases.^[18,19]^ In this current study, 3 sessions of acupuncture on alternating days were also tailored to the patient's willingness. We found that the mean perfusion value of the facial area also decreased during acupuncture stimulus and local area microcirculation in rosacea redistributed after acupuncture, suggesting the tentative therapeutic effectiveness of acupuncture for rosacea. The therapeutic effectiveness of rosacea treatment by acupuncture presented here is also in line with other studies. More specifically, it is well-documented that acupuncture treatment reduces itching and urticaria in patients with atopic eczema,^[10]^ and acupuncture-related treatment has been shown to significantly improve psoriasis symptoms,^[11]^ suggesting that acupuncture is a potentially effective alternative therapy for certain skin diseases such as rosacea, atopic eczema, and psoriasis.^[10,11]^

The specific mechanism of acupuncture's physiologically action relating to the formation of new microvasculature remains unknown. A previous study showed that the local blood flow of rosacea-affected skin decreased after intense pulsed light treatment, which improved the symptoms of erythema.^[20]^ Other studies also showed that improvement of blood flow in local affected areas was related to improvements of skin symptoms in some skin diseases such as psoriasis^[13]^ and ulcers.^[15]^ A recent study evaluated skin perfusion after acupuncture in atopic eczema patients, and the result showed that the mean perfusion value, as well as the mean wheal and flare size was significantly smaller in acupuncture group when compared with the nonacupuncture control group.^[10]^ At the medicinal level, brimonidine, a selective α2 adrenergic receptor agonist, was topically used for treatment of facial flushing and erythema caused by rosacea. Treatment with brimonidine resulted in vascular constriction that was confirmed by quantitative measurements and analyses.^[21]^ These studies indicate that modulating vascular circulation is important in the treatment of rosacea, and therapeutic effectiveness of acupuncture for rosacea might possibly be related to improvement in blood flow.

Although pathophysiology of rosacea is still poorly understood, cutaneous neurogenic inflammation is a factor that correlates with morphologic characteristics of erythematotelangiectatic, papulopustular, and ocular rosacea.^[1–3]^ These subtypes present with symptoms such as facial erythema, telangiectases, papules, and pustules.^[22–24]^ And one of the underlying mechanisms of acupuncture is cutaneous neurogenic inflammation changes at acupoints.^[25–27]^ Thus, these studies suggested that acupuncture could be possibly used to treat the papulopustular and ocular rosacea. However, the possibility of using acupuncture for these types of rosacea needs to be further explored.

In conclusion, our results demonstrate that acupuncture has the potential to be an alternative therapy for rosacea localized to the face, and the condition of perfusion distribution under the skin may relate to the severity of rosacea symptoms. Further studies using sample randomization and controlled trials with larger samples are needed to verify this finding and to optimize treatment protocols for rosacea.

## 9 Patient perspective

I had been suffered from itching, papules, and pustules repeatedly for about 18 months, and it worsened after I ate greasy foods. I felt embarrassed and anxious because of the redness, red rash, and increased sebum excretions from my nose, which affected my social activity and my life. I had used metronidazole cream, antifungal drugs, and steroidal ointments during those 18 months, but the effectiveness was poor. So, I stopped these treatments and decided to try acupuncture treatment. After 3 treatments of acupuncture, the erythema and papules of my nose were eliminated, and I felt much better about the appearance of my nose. Now, 6 months have passed since the acupuncture treatment and there has been no relapse. I am satisfied with my current condition.

## Author contributions

Gao YC managed the case with full responsibility. Gao YC and Chen YC wrote the manuscript. Lin WP and Zhou SS measured perfusion images. He J and Chen YC oversaw the treatment and revised the manuscript.

**Data curation:** Weipeng Lin.

**Software:** Sisi Zhou.

**Writing – original draft:** Yacen Gao.

**Writing – review & editing:** Guoqi Shi, Jun He, Yongjun Chen.
